# Effects of Combining Biofactors on Bioenergetic Parameters, Aβ Levels and Survival in Alzheimer Model Organisms

**DOI:** 10.3390/ijms23158670

**Published:** 2022-08-04

**Authors:** Lukas Babylon, Fabian Schmitt, Yannik Franke, Tim Hubert, Gunter P. Eckert

**Affiliations:** Biomedical Research Center Seltersberg (BFS), Laboratory for Nutrition in Prevention and Therapy, Institute of Nutritional Sciences, Justus Liebig University Giessen, Schubertstr. 81, 35392 Giessen, Germany

**Keywords:** Alzheimer disease, mitochondria, mitochondria dysfunction, folic acid, vitamin B6, magnesium-orotate, amyloid beta, *C. elegans*, biofactor

## Abstract

Increased amyloid beta (Aβ) levels and mitochondrial dysfunction (MD) in the human brain characterize Alzheimer disease (AD). Folic acid, magnesium and vitamin B6 are essential micro-nutrients that may provide neuroprotection. Bioenergetic parameters and amyloid precursor protein (APP) processing products were investigated in vitro in human neuroblastoma SH-SY5Y-APP695 cells, expressing neuronal APP, and in vivo, in the invertebrate *Caenorhabditis elegans* (CL2006 & GMC101) expressing muscular APP. Model organisms were incubated with either folic acid and magnesium-orotate (ID63) or folic acid, magnesium-orotate and vitamin B6 (ID64) in different concentrations. ID63 and ID64 reduced Aβ, soluble alpha APP (sAPPα), and lactate levels in SH-SY5Y-APP695 cells. The latter might be explained by enhanced expression of lactate dehydrogenase (LDHA). Micronutrient combinations had no effects on mitochondrial parameters in SH-SY5Y-APP695 cells. ID64 showed a significant life-prolonging effect in *C. elegans* CL2006. Incubation of GMC101 with ID63 significantly lowered Aβ aggregation. Both combinations significantly reduced paralysis and thus improved the phenotype in GMC101. Thus, the combinations of the tested biofactors are effective in pre-clinical models of AD by interfering with Aβ related pathways and glycolysis.

## 1. Introduction

At present, 50 million people are suffering from Alzheimer’s disease (AD) and this number will rise to approximately 152 million in 2050 [1]. Unfortunately, there is no cure for AD yet. Approved drugs only treat symptoms [2]. There are several hypotheses regarding the etiology of Alzheimer’s disease, but the causes of the disease are unknown. Previous research has focused on amyloid and tau, which has not yet led to major breakthroughs. Therefore, there is a trend towards multifactorial treatments and, among other things, energy metabolism with regard to mitochondrial functions. Two of the hallmarks of AD are mitochondrial dysfunction (MD) [3] and overproduction of beta-amyloid (Aβ) [4]. The first signs of beginning MD are a reduction of glucose consumption [5] and a reduced activity of key enzymes of the oxidative metabolism [6,7]. Almost all mitochondrial functions are impaired in AD [8,9]. The limited function of the electron transport chain (ETC) is the reason for the decrease in complexes IV and I. This results in a decreased mitochondrial membrane potential (MMP) and ATP production [10]. Another important characteristic of AD is that Aβ is cleaved of from a much larger amyloid precursor protein (APP) [11]. APP is cleaved via two pathways, a non-amyloid and an amyloidogenic pathway. The APP is spliced by the different types of protease, namely α-, β- and γ-secretase [11,12]. Depending on which protease cleaves the APP, Aβ peptides are produced. The α-protease cleaves the APP closer to the membrane, resulting in a shorter fragment in the membrane, which is then further cleaved by the γ-protease to a non-amyloidogenic product. However, when β-protease cleaves the protein, larger fragments are produced, which are then cleaved by γ-protease to form different large Aβ proteins [12] including Aβ_1–40_ and Aβ_1–42_ which may be the main triggers for AD [2,11,12]. It appears that Aβ_1–42_ has the greater neurotoxic potential compared to the Aβ_1–40_ form. Furthermore, Aβ_1–42_ tends to aggregate more, which can lead to plaque formation. In addition, the relationship between Aβ_1–40_ and Aβ_1–42_ is important, as the two influence each other [13,14,15]. There is evidence that sAPPα and sAPPβ share some properties [16], where sAPPα can be neuroprotective, whereas sAPPβ lacks most of the neuroprotective properties and has a rather negative effect [17]. Another early sign of AD is impaired glucose metabolisation which leads to MD and an increase in oxidant production [18,19]. The glycolysis pathway represents a way to ensure sufficient energy production and bypass Aβ induced impairment of mitochondria [20,21]. The enzymes pyruvate dehydrogenase kinase 1 (PDK1) and LDHA are of interest here. PDK1 phosphoylates pyruvate dehydrogenase and inactivates it. LDHA converts pyruvate into lactate. Both are markers of aerobic glycolysis. If a change occurs here, conclusions can be drawn about energy production [22,23].

There is evidence that specific biofactors [24], which are defined as substances required by the body for its normal physiological functioning and/ or with health-beneficial and/ or disease-preventive biological activities, may interfere with pathophysiological processes leading to AD [25,26,27]. A cocktail containing some of our tested compounds had a positive effect on AD symptoms in a TgF344-AD rat model. This could raise the mitochondrial function of the transgenic rats to the level of the wild-type rats [28]. Here, folic acid, magnesium-orotate and vitamin B6 in different combinations were tested in cellular and an invertebrate model of AD. The synthetically produced water-soluble folic acid, which consists of pterin, p-aminobenzoic acid and L-glutamic acid, belongs to the vitamin B complexes. Folate (also known as vitamin B9) is used as an umbrella term for the various derivatives of tetrahydrofolate (THF), with the synthetically produced form being referred to as folic acid [29,30]. Folate deficiency impairs DNA as well as mtDNA synthesis and stability and causes oxidative stress in the form of ROS, which, as already listed, is also associated with AD pathogenesis. In this context, neuronal impairment and increased cell death occur in AD. In addition, a deficiency of folate leads to a decrease in the methylation of enzymes and promoter regions of genes that are presumably also involved in AD pathogenesis [31,32]. Magnesium-orotate (MgOr), which is very poorly soluble in water, is the magnesium salt of orotic acid. As a source of magnesium (Mg), MgOr is used for the oral treatment of Mg deficiency. Orotic acid is a key intermediate in the biosynthetic pathway of pyrimidines and improves energy status by stimulating, among other things, the synthesis of glycogen and ATP [33]. Mg^2+^ is the fourth most abundant mineral as well as the second most abundant intracellular divalent cation in the human body and acts as a cofactor [34,35,36]. Mg is involved in protein synthesis, cellular energy production and storage, reproduction, DNA and RNA synthesis, and mitochondrial membrane potential [35]. Mg is also involved in the maintenance of physiological nerve and muscle function, cardiac excitability, and neuromuscular conduction [35,36]. Several pathological mechanisms in AD are discussed on which Mg might have a positive influence. Mg appears to inhibit the activity of γ-secretase and the proinflammatory TNF-α (tumor necrosis factor α) produced by microglia. Mg also inhibited IL-1β (interleukin-1β) and Aβ-induced, which all together induced inflammation. In addition, Mg has been reported to decrease the influx of Aβ across the blood-brain barrier [34]. High Mg concentrations have been shown to promote APP processing towards α-secretase due to the upregulation of transcription factors such as CREB [37]. Mg deficiency may be a risk factor for ADs and that possible supplementation may be a potentially valuable adjunct treatment for AD [38]. The water soluble vitamin B6 (Vit B6) is used as an umbrella term for various derivates from which pyridoxine is the most common form. It is an enzymatic cofactor required for more than 140 biochemical reactions, including transaminations, α-decarboxylations and replacement reactions [39]. Through the application of Vit B6, the oxidative stress induced by Aβ could be inhibited [40]. Furthermore, Vit B6 reduces the plasma levels of Aβ [41] and prevents the grey matter atrophy related to AD [42].

The present work investigated the effects of different B vitamins and MgOr on MD and the processing of APP in SH-SY5Y-APP_695_ cells a cellular model of early AD. Furthermore, the effects of the substances were tested in CL2006 and GMC101, both invertebrate models of AD.

## 2. Results

### 2.1. General Overview of Tests and Results

In Table 1 below, all tests and results are listed to provide a general overview of the subsequent tests and results. Here, the substance under investigation is shown against the control. For more detailed insights, the results are described in the respective chapters.

### 2.2. Aβ_1–40_ Production

First, we tested different concentrations of biofactors on Aβ_1–40_ production in SH-SY5Y-APP_695_ cells. Cells were incubated for 24 h with zinc orotate (ZnO), magnesium-orotate (MgO), benfotiamine (vitamin B1), folic acid (Fol), cholecalciferol (Vit D3), cobalamin (Vit B12), and pyridoxine (Vit B6) to select possible hit substances for further experiments (data not shown). Potential hit substances were identified, from which finally Fol 10 µM, MgOr 200 µM and Vit B6 100 nM turned out to be the most promising ones, which we applied in two different combinations.

**Figure 1:**
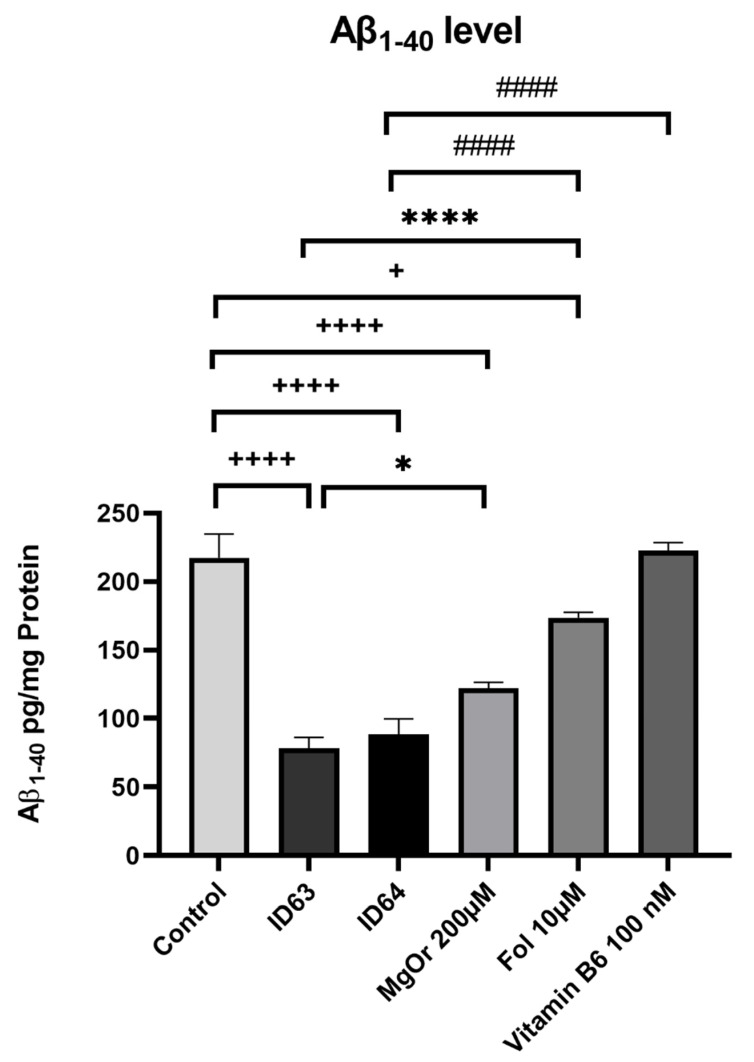
Effect of ID63 and ID64 in SH-SY5Y_695_ cells compared to the control or their single substances on the Aβ_1–40_ level after 24 h incubation. N = 6. Aβ_1–40_ levels were adjusted to the protein content. Significance was determined by Student’s unpaired *t*-test and one-way ANOVA. + significant against control; * significant against ID63; # significant against ID64. * *p* < 0.05, **** *p* < 0.0001, ^####^ *p* < 0.0001 and + *p* < 0.05, ^++++^ *p* < 0.0001. Data are displayed as the mean ± SEM. ID63 = 200 µM MgOr and 10 µM Fol; ID64 = 200 µM MgOr, 10 µM Fol and 100 nM Vit B6.

To investigate the effect on the Aβ_1–40_ production, SH-SY5Y-APP_695_ cells were incubated with both combinations (MgOr 200 µM & Fol 10 µM = ID63//MgOr 200 µM & Fol 10 µM & Vit B6 100 nM = ID64) and the single compounds for 24 h (Figure 1). ID63 had a significant lowering effect on the Aβ_1–40_ level (*p* > 0.0001). The ID63 combination even had an over additive effect compared to the single substances MgOr (*p* = 0.0028) and Fol (*p* > 0.0001). The ID64 combination had a significantly decreasing effect on the Aβ levels compared to the control (*p* > 0.0001). Furthermore, ID64 had a significantly reducing effect in comparison to the single substances MgOr (*p* = 0.0497), Fol (*p* > 0.0001) and Vit B6 (*p* > 0.0001).

### 2.3. Aβ_1–42_ Production

**Figure 2:**
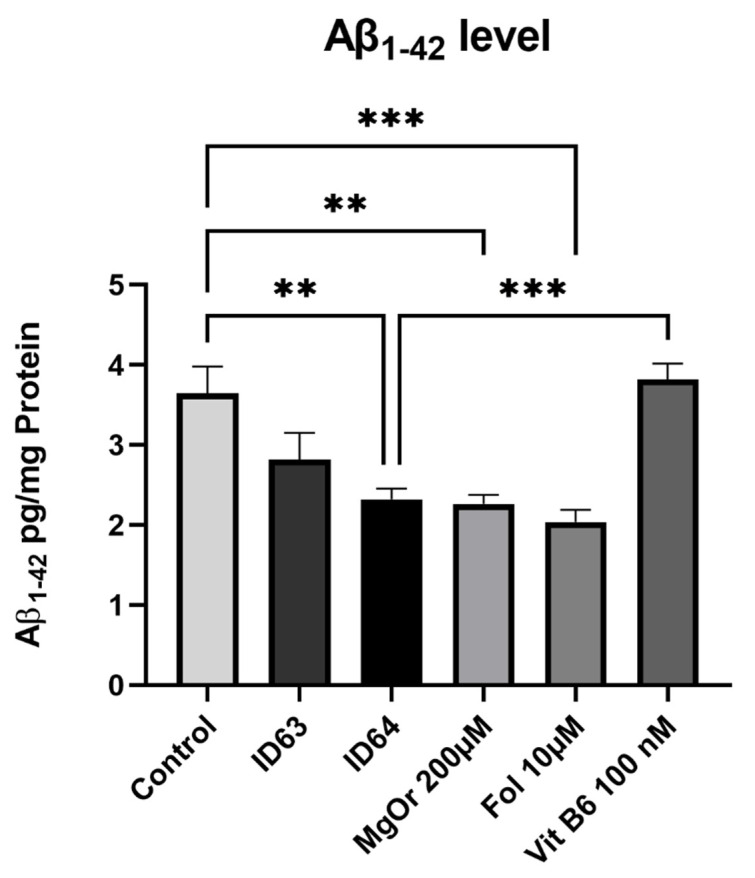
Effect of ID63 and ID64 in SH-SY5Y_695_ cells compared to the control or their single substances on the Aβ_1–42_ level after 24 h incubation. N = 6. Aβ_1–42_ levels were adjusted to the protein content. Significance was determined by Student’s unpaired *t*-test and one-way ANOVA. ** *p* < 0.01 and *** *p* < 0.001. Data are displayed as the mean ± SEM. ID63 = 200 µM MgOr and 10 µM Fol; ID64 = 200 µM MgOr, 10 µM Fol and 100 nM Vit B6.

To study the production of Aβ_1–42_, SH-SY5Y-APP_695_ cells were incubated for 24 h with ID63, ID64 or the single compounds (Figure 2). In comparison to the control, the single compounds MgOr (*p* = 0.0024) and folic acid (*p* = 0.0004), as well as the combination ID 64 (*p* = 0.0039) had a significant lowering effect on Aβ_1–42_ levels, while the combination ID63 had a slight reducing effect on the Aβ_1–42_ levels, although not a significant one. ID64 also showed significantly lower Aβ_1–42_ levels in comparison to B6 (*p* = 0.0009). However, folic acid alone, reduced the levels to a higher extent than any combinations.

### 2.4. sAPPα and sAPPβ Level

**Figure 3:**
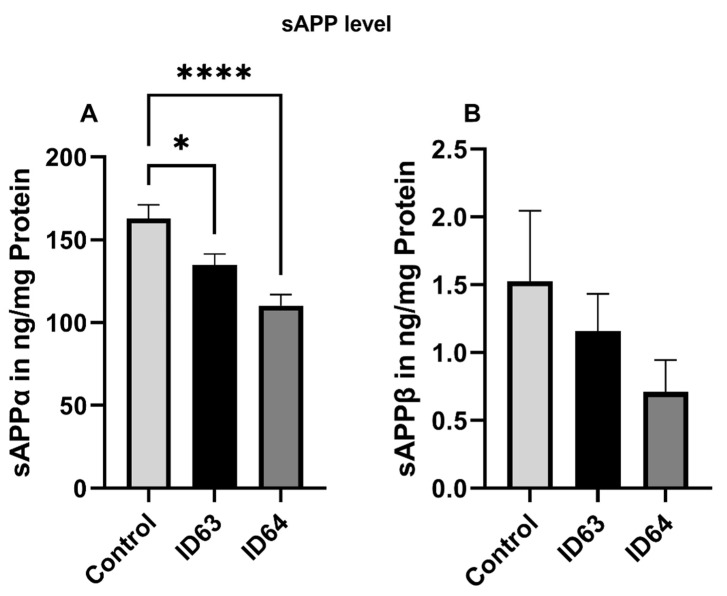
Effect of the incubation with ID63 or ID64 on the human soluble amyloid precursor protein α (sAPPα) and β (sAPPβ) after 24 h of incubation. (**A**) sAPPα level of SH-SY5Y_695_ cells after the incubation with ID63 or ID64 compared to the control. (**B**) sAPPβ level of SH-SY5Y_695_ cells after the incubation with ID63 or ID64 compared to the control. N = 6. sAPP levels were adjusted to the protein content. Significance was determined by one-way ANOVA. * *p* < 0.05 and **** *p* < 0.0001. Data are displayed as the mean ± SEM. ID63 = 200 µM MgOr and 10 µM Fol; ID64 = 200 µM MgOr, 10 µM Fol and 100 nM Vit B6.

**Figure 4:**
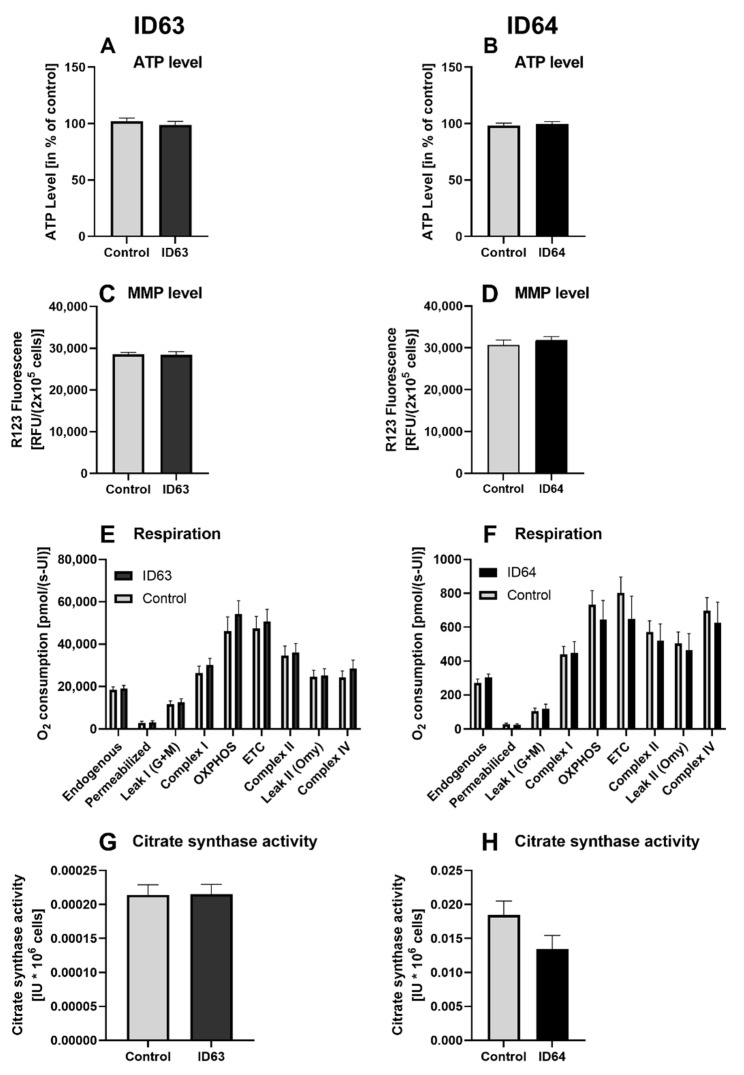
ATP level, MMP level, respiration and citrate synthase activity of SH-SY5Y-APP_695_ cells incubated for 24 h with ID63 or ID64. (**A**) ATP level of SH-SY5Y-APP_695_ cells incubated with ID63 and (**B**) ATP level of incubation with ID64 compared to the control. Cells treated with cell culture medium served as control (100%). N = 12. (**C**) MMP level of 2 × 10^5^ SH-SY5Y-APP_695_ cells incubated with ID63 and (**D**) MMP level of incubation with ID64 compared to the control. N = 16. (**E**) Respiration of SH-SY5Y-APP_695_ cells incubated with ID63 and (**F**) incubation with ID64 compared to the control. SH-SY5Y-APP_695_ cells adjusted to international units (IU) of citrate synthase activity. N = 15. (**G**) Citrate synthase activity of SH-SY5Y-APP_695_ cells incubated with ID63 and (**H**) Citrate synthase activity incubated with ID64 compared to control. N = 12. Significance was determined by Student’s unpaired *t*-test. Data are displayed as the mean ± SEM. ID63 = 200 µM MgOr and 10 µM Fol; ID64 = 200 µM MgOr, 10 µM Fol and 100 nM Vit B6. Leak I (G + M) = leak respiration with glutamate and malate; OXPHOS = oxidative phosphorylation system; ETC = electron transport chain; Leak II (Omy) = leak respiration with olygomycin.

The α- and β-secretase cleaving products of APP, sAPPα (Figure 3A) and sAPPβ (Figure 3B), respectively, were determined after incubation with either ID63 or ID64 for 24 h. Figure 4A shows that ID63 had a significantly lowering effect on the sAPPα (*p* = 0.0214) compared to the control. ID64 had an even greater effect on the reduction of sAPPα fragments (*p* > 0.0001). In contrast to the sAPPα fragment production, the sAPPβ fragments (Figure 3B) were lowered compared to the control though not significantly. ID64 had a greater effect than ID63. It should be noted that basal levels of sAPPβ were approximately one hundredfold lower compared with sAPPα (Figure 3).

### 2.5. Effect on the Mitochondrial Function

To investigate the effect of ID63 and ID64 on mitochondrial function, we incubated SH-SY5Y-APP_695_ cells for 24 h with ID63 or ID64. Respiration under O_2_ consumption through the respiratory chain builds up the mitochondrial membrane potential, which allows ATP to be generated with the help of ATP synthase. First, we measured the ATP level after incubation with ID63 or ID64. Afterwards, the MMP was examined as well as the O_2_ consumption and citrate synthase activity (Figure 4).

**Figure 5:**
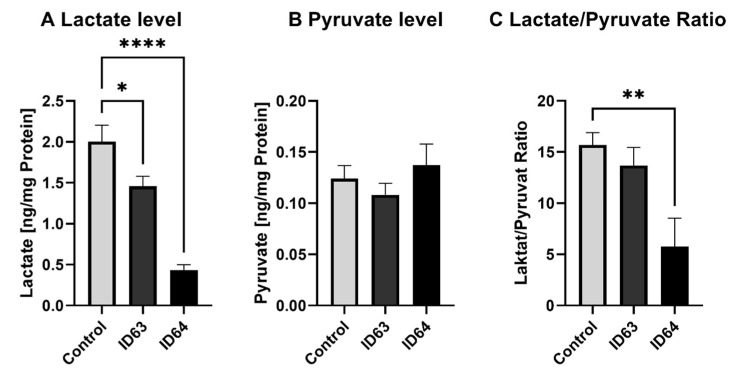
Effect of the incubation with ID63 or ID64 on lactate and pyruvate level after 24 h of incubation. (**A**) Lactate level of SH-SY5Y_695_ cells after the incubation with ID63 or ID64 compared to the control. (**B**) Pyruvate level of SH-SY5Y_695_ cells after the incubation with ID63 or ID64 compared to the control. (**C**) Lactate to pyruvate ratio. N = 6. Levels were adjusted to the protein content. Significance was determined by one-way ANOVA. * *p* < 0.05, ** *p* < 0.01 and **** *p* < 0.0001. Data are displayed as the mean ± SEM. ID63 = 200 µM MgOr and 10 µM Fol; ID64 = 200 µM MgOr, 10 µM Fol and 100 nM Vit B6.

Neither ID63 nor ID64 had an increased effect on the ATP level (Figure 5A,B) or an effect on the MMP level (Figure 4C,D). ID63 had a slightly increasing effect on the complex activity of complex I, II and IV compared to the control (Figure 4E). In contrast, ID64 had a slightly decreasing effect on the complex activity of complex II and IV (Figure 4F). Whereas ID63 had no effect on the citrate synthase activity compared with the control (Figure 4G), and ID64 even had a slightly decreasing effect compared with the control (Figure 4H). However, none of these effects is statistically significant.

### 2.6. Lactate and Pyruvate Level

**Figure 6:**
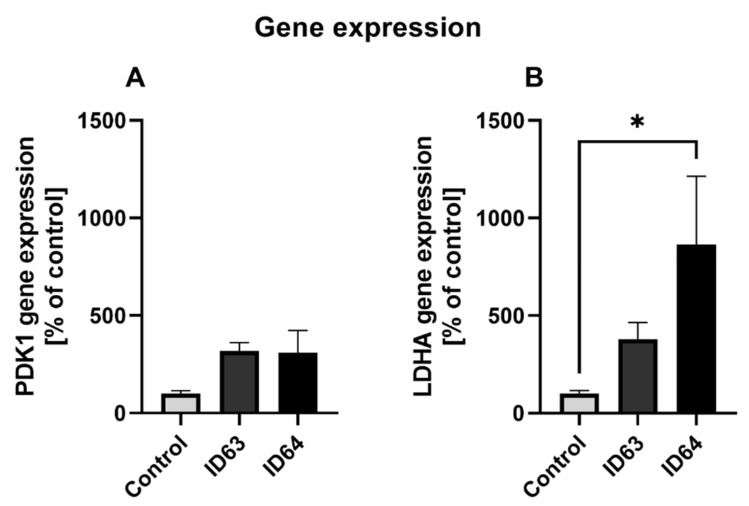
Effect of the incubation with ID63 or ID64 on the gene expression after 24 h of incubation. (**A**) Gene expression of pyruvate dehydrogenase kinase 1 (PDK1) of SH-SY5Y_695_ cells after the incubation with ID63 or ID64 compared to the control. (**B**) Gene expression of lactate dehydrogenase A (LDHA) of SH-SY5Y_695_ cells after the incubation with ID63 or ID64 compared to the control. N = 8. Significance was determined by one-way ANOVA. * *p* < 0.05. Data are displayed as the mean ± SEM. ID63 = 200 µM MgOr and 10 µM Fol; ID64 = 200 µM MgOr, 10 µM Fol and 100 nM Vit B6.

To investigate if the glycolysis is affected by ID63 or ID64, the lactate and pyruvate levels were measured. As seen in Figure 6, only lactate is significantly reduced by ID63 (*p* = 0.0328) and ID64 (*p* > 0.0001) compared to the control while pyruvate levels were not influenced. The ratio of lactate/pyruvate was significantly affected by ID64 (*p* = 0.0057) (Figure 5C).

### 2.7. qPCR

To investigate the molecular basis of altered lactate and pyruvate levels, the gene expression of pyruvate dehydrogenase kinase 1 (PDK1) and lactate dehydrogenase A (LDHA) were examined after 24 h incubation using qRT-PCR. ID63 and ID64 had no significant effect on PDK1 gene expression compared to the control (Figure 6). Both combinations increased LDHA mRNA levels (Figure 6B), with ID64 (*p* = 0.0148) showing a significant increase in gene expression.

### 2.8. Effect on the Lifespan of C. elegans in Heat Stress Survival Assay

**Figure 7:**
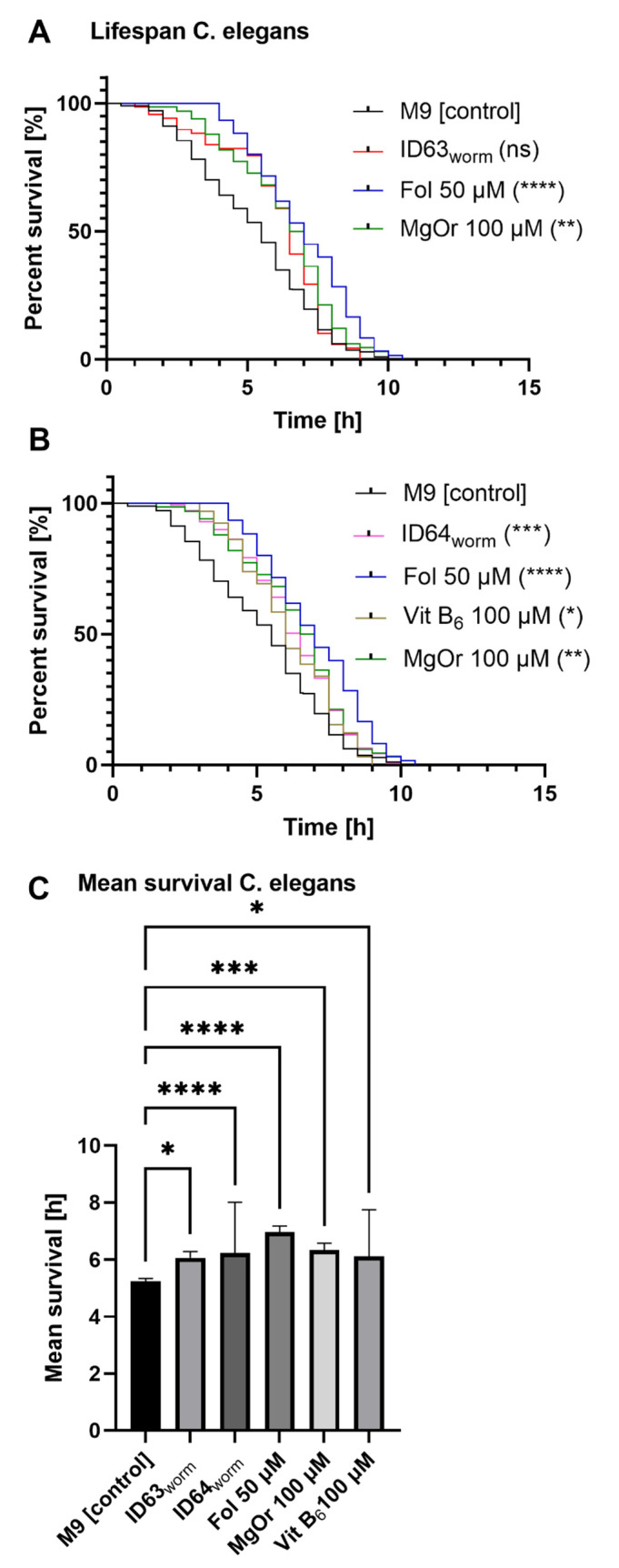
The lifespan under heat-stress of *C. elegans* after treatment with either ID63_worm_, ID64_worm_ or their single substances in CL2006. (**A**) The single substances lead to an increase in heat-stress resistance of the nematodes although the combination of them does not have a significant impact. (**B**) After combination with vitamin B_6,_ the resulting treatment leads to a significant increase in heat-stress resistance. For heat-stress experiments, the survival was assessed according to the penetration of SYTOX Green nucleic acid stain into dead cells. N > 60. log-rank (MantelCox) test. (**C**) Mean Survival of *C. elegans* after treatment with either ID63_worm_, ID64_worm_ or their single substances in CL2006. Significance was determined by one-way ANOVA. *p* * < 0.05, *p* ** < 0.01, *p* *** < 0.001 and *p* **** < 0.0001. ID63_worm_ = Fol 50 µM and MgOr 100 µM; ID64_worm_ Fol 50 µM, MgOr 100 µM and Vit B6 100 µM.

To test the effect of the combinations in vivo two invertebrate AD-models were used. *C. elegans* CL2006 were incubated with the same compounds but in different concentrations. The single compounds Fol 50 µM (*p* > 0.0001), Vit B6 100 µM (*p* = 0.0397) and MgOr 100 µM (*p* = 0.001) had a significant life-extending effect compared to the control (see Figure 7A,B). As shown in Figure 7A, the ID63_worm_ extended lifespan of CL2006 compared to the control by trend (*p* = 0.0502), whhereas in Figure 7B, it can be seen that ID64_worm_ had a significant life-prolonging effect (*p* = 0.0002) compared to the control. Subsequently, the mean survival of the nematodes after the incubations of the combinations and single substances was assessed. Thereby Figure 7C shows that ID63_worm_ (*p* = 0.0196), Fol 50 µM (*p* < 0.0001), MgOr 100 µM (*p* = 0.0004), ID64_worm_ (*p* < 0.0001) and Vit B6 (*p* = 0.0107) had a significant increasing effect on the mean survival of the nematodes compared to the control. Folic acid alone was numerically more effective than the combinations or the other single compounds.

### 2.9. Effect on the Paralysis

**Figure 8:**
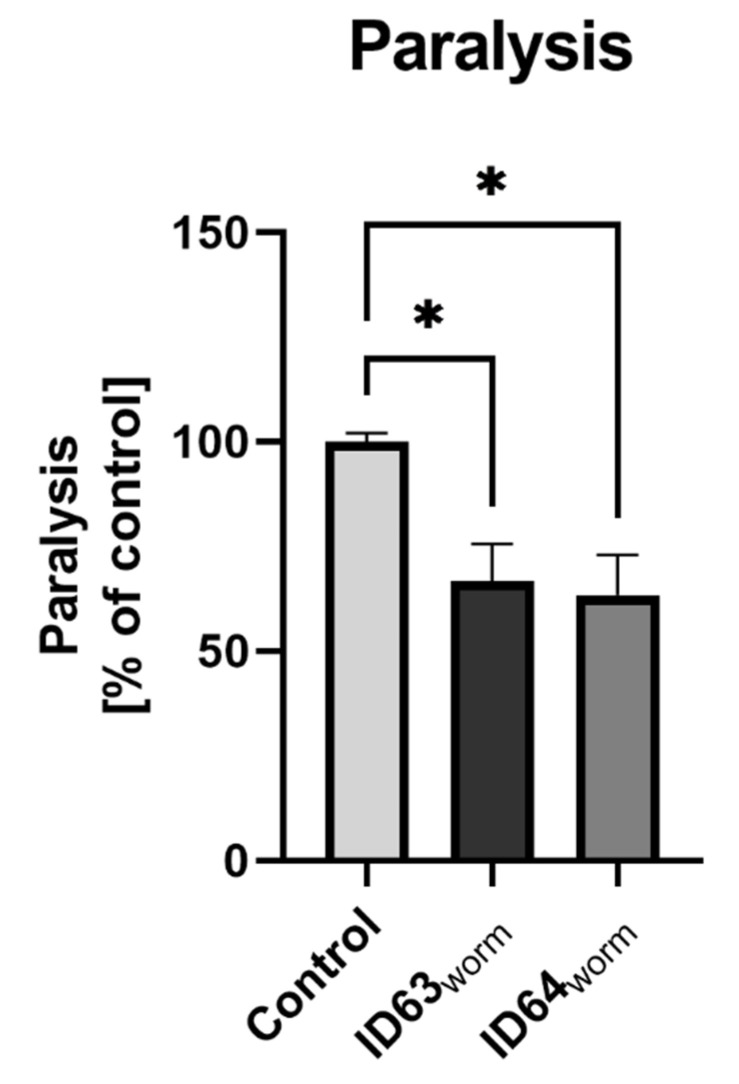
The Aβ induced paralysis after treatment with either ID63_worm_ or ID64_worm_ in GMC101. Both the feeding of both treatment combinations leads to significant reduction of Aβ induced paralysis after incubation of the nematodes for 24 h at 25 °C. N = 4. Mean ± SEM. One-way ANOVA with Tukey’s comparison post hoc test. *p* * < 0.05 ID63_worm_ = Fol 50 µM and MgOr 100 µM; ID64_worm_ Fol 50 µM, MgOr 100 µM and Vit B6 100 µM.

To investigate the effect of combining biofactors on paralysis induced by Aβ, *C. elegans* GMC101 were incubated for 24 h at 25 °C with either ID63_worm_ or ID64_worm_. Both combinations (*p* = 0.0243 for ID63_worm_ and *p* = 0.0149 for ID64_worm_) were able to significantly decrease the paralysis induced by Aβ (Figure 8). Thus, the phenotype of this AD-worm was significantly enhanced by both biofactor combinations to a comparable extent.

### 2.10. Aβ_1–42_ Production in GMC101

**Figure 9:**
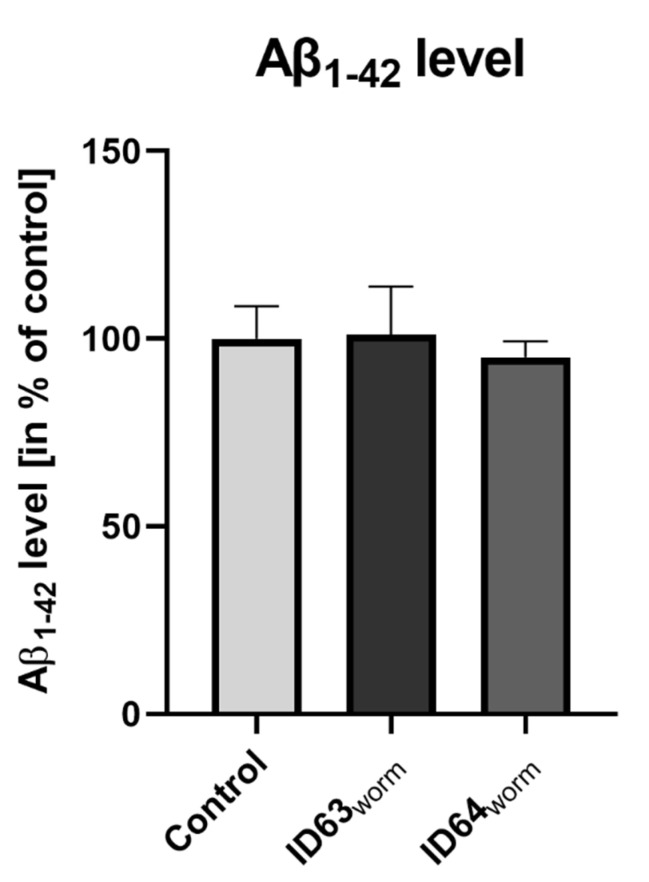
Effect of ID63 and ID64 in transgene nematodes GMC101 compared to the controls on the Aβ_1–42_ level after 48 h incubation. Neither ID63 nor ID64 did not lead to a significant alteration of Aβ_1–42_. N = 8. Aβ_1–42_ levels were adjusted to the protein content. N = 8. Mean ± SEM. Student’s *t*-test. ID63_worm_ = Fol 50 µM and MgOr 100 µM; ID64_worm_ Fol 50 µM, MgOr 100 µM and Vit B6 100 µM.

To study the production of Aβ_1–42_ in nematodes, GMC101 was incubated for 24 h at 25 °C with both combinations and then the Aβ level was examined. There was no effect on the Aβ_1–42_ levels compared to the control (Figure 9). Since Aβ_1–40_ is not produced in GMC101 this amyloid peptide was not investigated.

### 2.11. Aβ_1–42_ Aggregation

**Figure 10:**
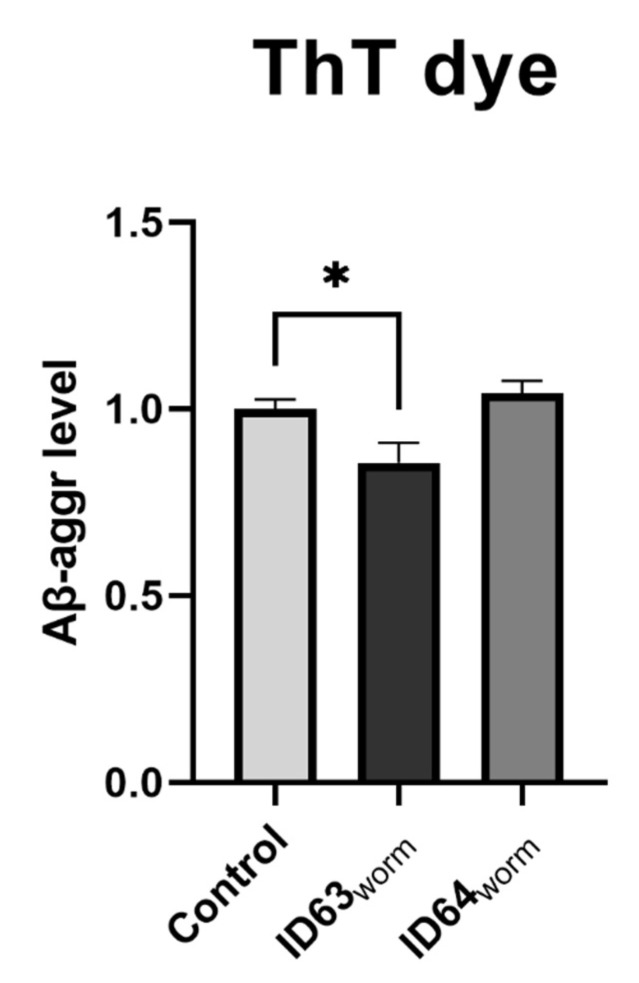
The effect of either ID63_worm_ or ID64_worm_ on the accumulation of aggregated Aβ (Aβ-aggr) in GMC101 after 48 h incubation. The treatment of the nematodes with ID63 leads to a significant reduction of accumulated Aβ. Whereby the treatment with ID64 combination did not lead to an alteration of Aβ levels; N = 8; mean ± SEM; one-way ANOVA with Tukey’s comparison post hoc test. * *p* < 0.05. ID63_worm_ = Fol 50 µM and MgOr 100 µM; ID64_worm_ Fol 50 µM, MgOr 100 µM and Vit B6 100 µM.

To investigate the aggregation of Aβ in nematodes, GMC101 was incubated at 25 °C for 24 h and stained with thioflavine (ThT), which labels β-sheet structures of aggregated peptides. There was a significant reduction of Aβ-aggr by the combination ID63_worm_ (*p* = 0.0324) compared to the control, whereas ID64_worm_ was without an effect (Figure 10).

## 3. Discussion

In the present work, we examined the effect of MgOr, Fol and Vit B6 in different combinations on bioenergetic parameters including mitochondrial function and glycolysis, as well as Aβ production in cellular and invertebrate models of AD. We wanted to create a combination product that achieves an optimal result by combining several biofactors. The hit compounds used in the current study were selected after a screening of seven substances of interest whose concentrations used in the experiments were based on known literature values [37,43,44,45,46,47]. We selected the concentrations because they were mostly tested in our cell model, related to AD or cell survival and they were used in relative physiological concentrations. This resulted in the combinations ID63 and ID64. The combinations ID63 and ID64 significantly reduced Aβ_1–40_ levels in SH-SY5Y-APP_695_ cells compared to the control (Figure 1). Even when compared to the individual components of the combinations, ID63 was able to show an over additively reducing effect on Aβ levels. Similar results were obtained by Li et al. who found a dose-dependent decrease of Aβ_1–40_ levels by incubation with Fol, modulating DNA methyltransferase activity [48]. In a study with AD patients, an intervention with Fol significantly reduced Aβ levels and increased the concentration of s-adenosyl methionine (SAM) [49]. Low SAM levels are a risk factor for AD, whereby incubation with SAM led to a decrease in Aβ levels in SK-N-SH cells [50]. Fol and Vit B6 are essential for the SAM cycle [51]. It seems that in our SH-SY5Y model the additional administration of Vit B6 shows no additional effect on Aβ_1–40_ levels. Furthermore, lower Mg^2+^ are associated with the occurrence of AD which negatively affects brain energy metabolism [52]. In addition, low Mg levels are also negatively correlated with the occurrence of Aβ_1–40_ and Aβ_1–42_ [53]. As a result, an administration of Mg^2+^ leads to a decrease in Aβ levels of N2a-APP cells [37]. After the incubation with ID63, SH-SY5Y-APP_695_ cells showed reduced levels of Aβ_1–42_ compared to the control, although not significantly. In contrast, the incubation with ID64 showed a significant reduction of Aβ_1–42_ (Figure 2) compared to the control. Administration of a Fol-rich diet significantly reduced Aβ_1–42_ levels in APP/PS1 mice compared to the standard diet [54]. Similarly, the incubation with Mg significantly decreased Aβ_1–42_ production in Na2 neuroblastoma cells and transgenic mice [37,55,56]. In our work, we could not reproduce these described effects shown despite the administration of both substances in SH-SY5Y-APP_695_ cells. However, it seems that Vit B6 has a crucial role in the reduction of Aβ_1–42_ levels (ID64) even if Vit B6 alone showed no effects. In contrast, it did not provide any benefit at the Aβ_1–40_ level (Figure 1). Next, we examined sAPPα and -β levels after incubation with ID63 and ID64. The reducing effects on Aβ_1–42_ levels by ID64 in SH-SY5Y-APP_695_ cells could not be confirmed in transgene nematodes GMC101. On the one hand, incubation decreased the sAPPα levels significantly compared to the control (Figure 3A). On the other hand, both ID63 and ID64 had a reducing but not significant effect on the sAPPβ levels compared to the control (Figure 3B). Whereas it should be noted that the concentration of sAPPβ is 100 times lower than sAPPα. Studies showed that the concentration of sAPPα is generally higher than that of sAPPβ [57,58]. An application of Mg^2+^ increased the amount of sAPPα in APP/PS1 transgenic mice and simultaneously decreased the concentration of sAPPβ [37]. The results shown in this study [37], where both sAPPα and -β were decreased, could not be reproduced in our work. We assume that this was due to a general decrease in APP production, since the application of Fol can reduce APP processing [59,60], which in turn can affect sAPPα and -β, resulting in generally lowered levels.

Next, we tested ID63 and ID64 on MD in SH-SY5Y-APP_695_ cells. SH-SY5Y-APP_695_ cells show reduced ATP level, MMP and O_2_ consumption compared to their non-transfected SH-SY5Y-MOCK cells [61]. Mg and Fol are vital compounds for enzymes and ATP production in cells [31,33,36]. A deficit leads to the reduced production of ATP [31,34]. In our case, which is not a deficit model, we did not observe any improvement in ATP level, MPP, O_2_ consumption or citrate synthase activity in SH-SY5Y-APP_695_ cells (Figure 4). In a study by Viel et al. using a similar cocktail consisting of some of our compounds, the mitochondrial complex activity in a transgenic rat model was increased to that of wild type rats [28]. This effect is not found in our case, possibly due to the additional substances contained in the cocktail, which had been the decisive factor here.

It has been shown that in the brains of AD patients there is a switch from aerobic respiration to glycolytic metabolism [62,63]. This is accompanied by an increase in lactate and pyruvate values [64,65], which results from insufficient utilization in oxidative phosphorylation [66]. Both biofactor combinations used in our investigations were able to significantly reduce the lactate values compared to the untreated control cells. The switch to increased oxidative phosphorylation could not be shown in our work, because there was no effect on ATP, MMP, OXPHOS or citrate synthase activity in the respiratory chain. To investigate the impact of the two combinations on the glycolytic genes, mRNA levels of PDK1 and LDHA were determined (Figure 6). There was an increase in the expression of PDK1 by both combinations compared to the control. PDK1 phosphorylates pyruvate dehydrogenase and inactivates it. This may lead to a reverse transport of pyruvate from the mitochondrion into the cytosol, where it is used for glycolytic energy production via lactate [67,68]. Both combinations were able to increase the expression of LDHA, while ID64 did so significantly. LDHA converts pyruvate to lactate and vice versa [23,69], although the expression was increased there were decreased lactate levels with both ID63 and ID64 (Figure 5). By upregulating PDK1 and LDHA expression, the cell may reduce the effects of Aβ toxicity and ROS production by shifting from mitochondrial to glycolytic energy production [23,69]. Both combinations appear to support this process, resulting in an upregulation of expression relative to control.

Based on the results obtained, especially those related to Aβ, we wanted to test our compounds in another model of AD. For this purpose, we adjusted the concentrations and tested them in two invertebrate models of AD. CL2006 and GMC101 both express human Aβ in their muscle cells. While CL2006 produces Aβ continuously at 20 °C [70], GMC101 needs a temperature shift to 25 °C to initiate Aβ production [71]. Phenotypically, both are identified like wild-type N2 [72]. ID63_worm_ had no significant life-prolonging effect on CL2006 compared to the control. Whereas the single substances and ID64_worm_ had a significant effect on the lifespan (Figure 7A,B). In contrast, all tested combinations and single substances, have a significantly increased effect on mean survival (Figure 7C). CL2006 continuously produces Aβ, which has a toxic effect on the lifespan [70,73]. In particular, it has effects on DAF-16, which is expressed in the nucleus during stress and has an effect on life span extension [74]. DAF-16 is notable for being responsible for activating genes involved in longevity, lipogenesis, heat shock survival and oxidative stress responses [75,76], homologs being found in *C. elegans*, humans and mice [77]. It was demonstrated that incubation with Fol extended the lifespan by increasing the expression of DAF-16 [78]. It seems that the substances used have a similar effect on lifespan, whereas the single substance Fol seems to be superior. Furthermore, it was investigated whether the substances can reduce the toxic effect caused by Aβ. For this purpose, it was examined whether the paralysis in GMC101 changes because of the administration (Figure 8). Both combinations were able to reduce the paralyzes, indicating the significantly reduced toxicity of Aβ. Similar effects have been shown with other substances, which reduced the paralysis and thus the toxicity of Aβ [73,79,80,81]. The reduced toxicity could be a consequence of the reduction of Aβ levels in *C. elegans*. It could be shown that the administration of single substances leads to a reduction of the Aβ levels [37,48,50,54,55]. To verify this, the Aβ_1–42_ levels were determined in GMC101 (Figure 9). Here, we found no significant effect in reduced Aβ level after incubation with both combinations. It should be noted here that the effects on Aβ_1–42_ were also relatively moderate in the cells (see above). Aβ_1–40_ was not measured in GMC101 because the worm does not produce these peptides. To examine the aggregation of Aβ after incubation with both combinations, samples were stained with ThT, which labels Aβ-structures in proteins (Figure 10). A significant reduction in Aβ-aggr was observed after incubation with ID63_worm_. The combination with additional vitamin B6 did not reduce aggregation further. A similar effect was found in a study by Yu et al., in which the aggregation of Aβ in mice was reduced by incubation with magnesium [82].

It remains to be considered that there are both advantages and disadvantages to administering the substances as a single or combination preparation. Combined administration could lead to synergistic effects that enhance the positive effects and thus lead to an additive effect, as shown in the Aβ_1–40_ values Figure 1. However, it cannot be ruled out that negative effects may occur when more than one substance is administered, as the substances may influence each other. Complex formation or competition for transport systems into the cell could occur. Especially when moving from an in vitro to an in vivo experiment. In order to exclude these effects, further experiments will have to be carried out in the future.

## 4. Materials and Methods

### 4.1. Cell Culture

The cultivation of SY5Y cells was performed in 75 cm^2^ cell culture flasks under sterile conditions. To ensure optimal growth, cells were split several times per week once a cell density of approximately 70–80% was reached. All cells were cultured in an incubator at 37 °C and 5% CO_2_ in DMEM supplemented with 10% (*v*/*v*) heat-inactivated fetal calf serum, 60 µg/mL streptomycin, 0.3 mg/mL hygromycin, 1% MEM non-essential amino acids, and 1 mM sodium pyruvate 1%, 60 units/mL penicillin. SH-SY5Y cells were stably transfected with DNA constructs harboring human wild-type APP_695_ (APP_695_) and were kindly donated by A. Eckert (Basel, Switzerland) (for details; please refer to [83]).

### 4.2. Cell Treatment

First of all, cells were incubated for 24 h with different concentrations of zinc orotate (ZnOr), magnesium-orotate (MgOr), benfotiamine (vitamin B1), folic acid (Fol), cholecalciferol (Vit D3), cobalamin (Vit B12), and pyridoxine (Vit B6) for possible hit substances. After the hit substances were determined, cells were incubated with 200 µM magnesium-orotate (MgOr) and folic acid 10 µM (Fol) (combination ID63) or 200 µM MgOr, 10 µM folic acid and 100 nM Vit B6 (combination ID64). We have derived the concentrations used here from the literature [37,43,44,45,46,47]. The solution medium NaOH diluted 1:8 with water served as a control. The ratio had the best dissolving properties without being toxic to the cells.

### 4.3. ATP Measurement

A bioluminescence assay was used to determine the ATP levels, which is based on the production of light from ATP and luciferin in the presence of luciferase. The test was performed using the ATPlite Luminescence Assay System (PerkinElmer, Rodgau, Germany) according to the previously published protocol [84].

### 4.4. MMP Measurement

Mitochondrial membrane potential (MMP) was measured using the fluorescence dye rhodamine-123 (R123). Cells were incubated for 15 min with 0.4 µM R123 and centrifuged at 750× *g* for 5 min before being washed with Hank’s Balanced Salt Solution (HBSS) buffer supplemented with Mg^2+^, Ca^2+,^ and HEPES. The cells were suspended with fresh HBSS before they were evaluated by measuring the R123 fluorescence. The excitation wavelength was set to 490 nm and the emission wavelength to 535 nm witch CLARIOstar (BMG Labtech, Ortenberg, Germany).

### 4.5. Cellular Respiration

Respiration in SH-SY5Y_695_ cells was assessed using an Oxygraph-2k (Oroboros, Innsbruck, Austria) and DatLab 7.0.0.2. The cells were treated according to a complex protocol developed by Dr. Erich Gnaiger [85]. They were incubated with different substrates, inhibitors and uncouplers. First, cells were washed with PBS (containing potassium chloride 26.6 mM, potassium phosphate monobasic 14.705 mM, sodium chloride 1379.31 mM and sodium phosphate dibasic 80.59 mM) and scraped into mitochondrial respiration medium (MiRO5) developed by Oroboros [85]. Afterwards, the cells were centrifuged, resuspended in MiRO5, and diluted to 10^6^ cells/mL. After 2 mL of cell suspension was added to each chamber and endogenous respiration was stabilized, the cells were treated with digitonin (10 µg/10^6^ cells) to permeabilize the membrane, leaving the outer and inner mitochondrial membrane intact. OXPHOS was measured by adding the complex I and II substrates malate (2 mM), glutamate (10 mM) and ADP (2 mM), followed by succinate (10 mM). Gradual addition of carbonyl cyanide-4- before it is evaluated by measuring the R123 fluorescence (trifluoromethoxy) phenylhydrazone showed the maximum capacity of the electron transfer system. Rotenon (0.1 mM) was added to measure the activity of complex II. To investigate the leak respiration, oligomycin (2 µL/mL) was injected. To inhabitation of complex III to determined residual oxygen consumption, antimycin A (2.5 µM) was added. This value was subtracted from all respiratory states. Adding N measured N, N′, N′-tetramethyl-p-phenylenediamine (0.5 mM) and ascorbate (2 mM) the activity of complex IV. To measure the sodium autoxidation rate, azide (≥100 mM) was added. Afterwards, complex IV respiration was corrected by subtracting the autoxidation rate of azid. NaOH served as control.

### 4.6. Citrate Synthase Activity

Cell samples from respirometry measurements were frozen and stored at −80 °C for the determination of citrate synthase activity. Samples were thawed while the reaction mix (0.1 mM 5,5′-dithiol-bis-(2-nitrobenzoic acid) (DTNB), 0.5 mM oxaloacetate, 50 μM EDTA, 0.31 mM acetyl coenzyme A, 5 mM triethanolamine hydrochloride, and 0.1 M Tris-HCl) was mixed and heated at 30 °C for 5 min. Afterwards, 40 µL of samples were submitted in triplets and mixed with 110 µL of the reaction mix. The absorption was measured at 412 nm.

### 4.7. Aβ_1–40_ Measurement

After 24 h incubation, the Aβ_1–40_ levels were determined in SH-SY5Y-APP_695_ cells using HTRF Amyloid-Beta 1–40 kit (Cisbio, Codolet, France). The protocol was the same as described earlier [61]. Aβ concentrations were normalized against protein content.

### 4.8. Aβ_1–42_ Measurement

After 24 h incubation, the Aβ_1–42_ levels were determined in SH-SY5Y-APP_695_ cells and nematodes GMC101 using the Human-Aβ_1–42_ ELISA Kit (Invitrogen^TM^, Waltham, MA, USA). The protocol was performed according to the manufacturer’s instructions. Aβ concentrations were normalized against protein content.

### 4.9. Protein Quantification

Protein content was determined using a Pierce^TM^ Protein Assay Kit (Thermo Fisher Scientific, Waltham, MA, USA) according to the manufacturer’s instructions. Bovine serum albumin was used as a standard.

### 4.10. Quantification of Human Soluble Amyloid Precursor Protein α (sAPPα)

The sample preparation was the same according to the Aβ_1–40_ quantification. The sAPPα levels were determined using the Human Soluble Amyloid Precursor Protein α (sAPPα) ELISA Kit (Cusabio, Wuhan, China). The process was carried out according to the manufacturer’s instructions. Levels were normalized to the protein content.

### 4.11. Quantification of Human Soluble Amyloid Precursor Protein β (sAPPβ)

The sample preparation was the same according to the Aβ_1–40_ quantification. The sAPPβ levels were determined using the Human Soluble Amyloid Precursor Protein β (sAPPβ) ELISA Kit (BT LAB, Zhejiang, China). The process was carried out according to the manufacturer’s instructions. Levels were normalized to the protein content.

### 4.12. Pyruvate and Lactate Content

Frozen cells, which were previously harvested and incubated for 24 h, were thawed at room temperature. Pyruvate and lactate concentrations were assessed using a pyruvate assay kit (MAK071, Sigma Aldrich, Darmstadt, Germany) and a lactate assay kit (MAK064, Sigma Aldrich, Darmstadt, Germany) according to the manufacturer’s instructions.

### 4.13. Real-Time qRT-PCR

To isolate RNA, we used the RNeasy Mini Kit (Qiagen, Hilden, Germany) according to the manufacturer’s guidelines. Nanodrop^TM^ 2000c spectrometer (Thermo Fisher Scientific, Waltham, MA, USA) was used to quantify RNA. The TURBO DNA-free^TM^ kit was used according to the manufacturer’s instructions (Thermo Fisher Scientific, Waltham, MA, USA) to remove residual genomic DNA. cDNA was synthesized from 1 µg total RNA using an iScript cDNA Synthesis Kit (Bio-Rad, Munich, Germany). qRT-PCR was conducted using a CfX 96 Connect™ system (Bio-Rad, Munich, Germany). All used primers are listed in Table 2. The cDNA aliquots were analyzed in triplicate and diluted 1:10 with RNase-free water (Qiagen, Hilden, Germany). PCR cycling conditions were as follows: initial denaturation for 3 min at 95 °C, followed by 45 cycles at 95 °C for 10 s, 58 °C for 30 s (or 56 °C for 45 s, depending on the primer), and 72 °C for 29 s. Expression was analyzed with −(2∆∆Cq) using Bio-Rad CfX manager software. To normalize the values a factor was calculated based on the geometric mean of the levels of multiple control genes of *ß-actin (ACTB), glyceraldehyde-3-phosphate dehydrogenase (GAPDH)*, and *phosphoglycerate kinase 1 (PGK1)* according to the MIQE guidelines [86]. RNA free water served as an assay control to exclude impurities.

### 4.14. Nematode and Bacterial Strain

*C. elegans* strains were obtained from the Caenorhabditis Genetics Center (University of Minnesota, MN, USA) and included CL2006 [Punc-54::human A-beta 3–42; pRF4 (rol-6(su1006))] and GMC101 [(Punc-54::A-beta::unc-54 3Prime UTR; Pmtl2::GFP)]. The strain GMC101 expresses the full-length human Aβ_1–42_ peptide in body-wall muscle cells that aggregates in vivo. Shifting L4 or young adult animals from 20 °C to 25 °C could induce the expression of Aβ and cause paralysis. The strain CL2006 constitutively expressed Aβ when cultured at 20 °C.

Nematodes were maintained on nematode growth medium (NGM) agar plates seeded with the bacterial *E. coli* strain OP50. According to standard protocols, the seeded plates were stored at 20 °C [87]. Synchronous populations were generated for all experiments by using a standard bleaching protocol [88].

### 4.15. Cultivation and Treatment

Post-bleaching generated larvae were washed twice in M9-buffer and the number of larvae in 10 µL were adjusted to 10 larvae. Afterward, the synchronized larvae were raised in cell culture flasks (Sarstedt, Nümbrecht, Germany) in either an amount of 1000 or 5000 nematodes, depending on the experiments. OP50-NGM was added to the flasks as a standardized source of food. The larvae were maintained under shaking at 20 °C until they reached young adulthood within 3 days. The micronutrients were dissolved in advance in M9 buffer. For each micronutrient observed in this study, we generated a series of concentrations as follows. Folic acid (Fol) 50 µM, magnesium orotate (MgOr) 0.1 mM (together ID63_worm_) and Fol 50 µM, MgOr 0.1 mM and vitamin B6 (Vit B6) 100 µM (together ID64_worm_). After reaching adulthood (48 h before the experiment), the micronutrients were added to the flasks. Pure M9-buffer was used as a control. Then, 24 h before the experiment amyloid aggregation was proceeded by upshifting young adult GMC101 from 20 °C to 25 °C.

### 4.16. Paralyze Assay

Cell culture flasks containing approximately 1000 adult amyloid beta producing nematodes were incubated for several hours to achieve Aβ induced paralysis (GMC101 24 h at 25 °C). On a NGM Agar Plate, 25 nematodes were placed and by physically touching with a platinum tip the paralysis status was recorded. Nematodes which normally act after being touched by the wire are recognized as “not paralyzed” whereas uncoordinated movements or just head movements were recorded as “paralyzed”.

### 4.17. Heat-Stress Survival Assay

After 48 h of incubation of CL2006 with the mentioned effectors, the time till death was determined using a microplate thermotolerance assay [89]. In preparation, the nematodes were washed out of the flasks with M9-buffer into 15 mL tubes followed by 3 additional washing steps. Each well of a black 384-well low-volume microtiter plate (Greiner Bio-One, Frickenhausen, Germany) was prefilled with 6.5 µL M9-buffer/Tween^®^20 (1% *v*/*v*). In the following step, one nematode in 1 µL M9-buffer was transferred and immersed in the well under a stereomicroscope (Breukhoven Microscope systems, Netherlands). SYTOX™ Green (Life Technologies, Karlsruhe, Germany) in a final concentration of 1 µM was added to reach a final volume of 15 µL in the well. SYTOX™ Green creates a fluorescent signal after binding to DNA. The plates were sealed with a Rotilab sealing film (Greiner Bio-One, Frickenhausen, Germany). The heat-shock was applied and the fluorescence was measured every 30 min for 17 h at 37 °C following the protocol previously described [90]. The excitation was set at 485 nm and the emission was detected at 538 nm.

### 4.18. ThT Dying of Aβ Aggregates

Detection and quantification of Aβ aggregates (Aβ-aggr) in GMC101 were performed using the fluorescent dye thioflavin T (ThT) according to a previously described method with minor modifications [91]. Synchronized and heat incubated nematodes were washed out of the cell culture flasks with M9-buffer/Tween^®^20 (1% *v*/*v*) and separated from larvae. After centrifugation, 200 µL of a thick pellet of nematodes was transferred into a microcentrifuge tube and were frozen in liquid nitrogen. Samples were thawed with 500 µL of PBS including proteinase inhibitor. Afterward, the samples were homogenized with a sonifier 3 × 20 s on ice. Protein contents in the homogenate were assessed according to the Pierce™ BCA Protein Assay Kit (Thermo Fisher Scientific, Waltham, MA, USA). Bovine serum albumin was used as a standard. Finally, fluorescence was measured in a black 96-well plate by adding 1 mM ThT (final concentration 20 mM). The volume in each well was 100 µL by adding M9. To determine the fluorescence of ThT, samples were measured by excitation at 440 nm and emission at 482 nm.

### 4.19. Statistics

Unless otherwise stated, values are presented as mean ± standard error of the mean (SEM). Statistical analyses were performed by applying one-way ANOVA with Tukey’s multiple comparison post-hock test, log-rank (Mantel-Cox) test and student’s unpaired *t*-test (Prism 9.1 GraphPad Software, San Diego, CA, USA). Statistical significance was defined for *p* values ns = not significant, * *p* < 0.05, ** *p* < 0.01, *** *p* < 0.001 and **** *p* < 0.0001.

## 5. Conclusions

In the present study, we reported that different combinations of folic acid, magnesium orotate, and vitamin B6 had significant effects on glycolysis, Aβ production, and Aβ aggregation in SH-SY5Y-APP_695_ cells and *C. elegans*. The phenotype of the in vivo model was significantly improved, highlighting the potential of the tested biofactor combinations as candidate therapeutics in AD. However, since the data did not consistently show a benefit of either combination, this study does not allow a clear statement as to whether vitamin B6 is required in addition to the combination of folic acid and magnesium orotate.

## Figures and Tables

**Table 1:** General overview of all tests and results of all biofactors. Combinations ID63 and ID64 and the single substances compared to the control.

	ID63 vs. CTR	ID64 vs. CTR	MgOr vs. CTR	Fol vs. CTR	Vit B6 vs. CTR
Aβ_1–40_	Significant lower	Significant lower	Significant lower	Significant lower	No significant change
Aβ_1–42_	No significant change	Significant lower	Significant lower	Significant lower	Significant lower
sAPPα	Significant lower	Significant lower	Not tested	Not tested	Not tested
sAPPβ	No significant change	No significant change	Not tested	Not tested	Not tested
ATP level	No significant change	No significant change	Not tested	Not tested	Not tested
MMP level	No significant change	No significant change	Not tested	Not tested	Not tested
Respiration	No significant change	No significant change	Not tested	Not tested	Not tested
Citrate synthase activity	No significant change	No significant change	Not tested	Not tested	Not tested
Lactate level	Significant lower	Significant lower	Not tested	Not tested	Not tested
Pyruvate level	No significant change	No significant change	Not tested	Not tested	Not tested
Lactate/Pyruvate Ratio	No significant change	Significant lower	Not tested	Not tested	Not tested
Gen expression PDK1	No significant change	No significant change	Not tested	Not tested	Not tested
Gen expression LDHA	No significant change	Significant higher	Not tested	Not tested	Not tested
Lifespan *C. elegans* in %	No significant change	Significant higher	Significant higher	Significant higher	Significant higher
Mean survival *C. elegans*	Significant higher	Significant higher	Significant higher	Significant higher	Significant higher
Paralysis *C. elegans*	Significant lower	Significant lower	Not tested	Not tested	Not tested
Aβ_1–42_ *C. elegans*	No significant change	No significant change	Not tested	Not tested	Not tested
Aβ_1–42_ aggreation *C. elegans*	Significant lower	No significant change	Not tested	Not tested	Not tested

**Table 2:** Oligonucleotide primer sequences, product sizes, and primer concentrations for quantitative real-time PCR.

Primer	Sequence	Manufacturer	Product Size	Concentration (nM)
ß-Actin (ACTB) NM_001101.2	5′-ggacttcgagcaagagatgg-3′ 5′-agcactgtgttggcgtacag-3′	Biomol, Hamburg, Germany	234	200
Glyceraldehyde-3-phosphate dehydrogenase (GAPDH) NM_002046.2	5′-gagtcaacggatttggtcgt-3′ 5′-ttgattttggagggatctcg-3′	Biomol, Hamburg, Germany	238	200
Phosphoglycerate kinase 1 (PGK1) NM_000291.2	5′-ctgtgggggtatttgaatgg-3′ 5′-cttccaggagctccaaa-3′	Biomol, Hamburg, Germany	198	200
Pyruvate dehydrogenase kinase, isozyme 1 (PDK1) NM_002610	5′-atacggatcagaaaccgaca-3′ 5′-cagacgcctagcattttcat-3′	Biomol, Hamburg, Germany	291	100
Human lactate dehydrogenase A like 6B (LDHA) NM_033195	5′-ggtgtccctttgaaggatct-3′ 5′-tgcagtcacttctttgtgga-3′	Biomol, Hamburg, Germany	87	400

## Data Availability

The data presented in this study are available on request from the corresponding author.
